# International expert perspectives on pharmacogenomics in pharmacy education: a qualitative study with implications for South Africa

**DOI:** 10.3389/fphar.2026.1888534

**Published:** 2026-08-18

**Authors:** Nicole Keuler, Jane McCartney, Rustin Crutchley, Renier Coetzee

**Affiliations:** 1 School of Pharmacy, University of the Western Cape, Cape Town, South Africa; 2 Ben and Maytee Fisch College of Pharmacy, University of Texas at Tyler, Texas, TX, United States; 3 School of Public Health, University of the Western Cape, Cape Town, South Africa

**Keywords:** competency, curriculum, experiential learning, pharmacist, pharmacogenomics, pharmacy education

## Abstract

**Introduction:**

Pharmacogenomics (PGx) has the potential to transform healthcare in South Africa through the delivery of precision medicine. However, the successful implementation of PGx services depends on a healthcare workforce equipped with appropriate PGx competencies. Given pharmacists’ central role in medication management, they are key members of the PGx team and require targeted education to support PGx-guided clinical decision-making. Insights from international experts can inform the development of pharmacy curricula, strengthening pharmacists’ capacity to facilitate PGx implementation within local healthcare systems.

**Method:**

A qualitative study design was used to explore international experts’ perspectives on the inclusion of PGx in pharmacy education. Health professional experts in PGx across the globe, including pharmacists from community and hospital settings, medical doctors, researchers, and clinical pharmacologists, were purposively sampled to participate in semi-structured interviews. The interviews explored views on PGx practices, the role of higher-education institutions and professional societies, and recommendations for PGx education across undergraduate, postgraduate, and continuing professional development pathways.

**Result:**

Twenty experts representing 12 countries were interviewed. Three overarching themes were identified: (1) PGx competency for pharmacists, (2) approaches to PGx education, and (3) barriers to PGx education. Subthemes included the evolving professional role of pharmacists in PGx, the knowledge and skills required for competent practice, and the distinction between foundational and advanced levels of PGx proficiency. Additional sub-themes addressed core PGx curricular content, effective instructional and assessment strategies, the role of community and stakeholder engagement, considerations regarding the appropriate depth, and integration of PGx material within pharmacy curricula.

**Conclusion:**

Experts internationally emphasized that PGx should be established as a core competency for all pharmacists. Pharmacists with both foundational and applied PGx competencies are well-positioned to contribute meaningfully to the clinical integration and advancement of PGx services. A longitudinal, integrated educational approach delivered by academics and health professionals with PGx expertise was viewed as essential for preparing students to apply PGx principles effectively in real-world practice. For South Africa, coordinated collaboration among higher education institutions, healthcare organizations, and professional regulatory bodies will be critical to embedding PGx within pharmacy education and advancing national precision-medicine goals.

## 1 Introduction

Africa continues to face a growing burden of communicable and non-communicable diseases (Vollset et al., 2024; Soko et al., 2023), underscoring the need for safe medicine-use practices. Pharmacogenomics (PGx) offers a promising pathway to improve patient health outcomes through rational use of medicine. A recent study showed that 16–54 medicines listed on African countries’ National Essential Medicine Lists have actionable pharmacogenes (Mazhindu et al., 2026), highlighting the potential for PGx to enhance patient safety and optimize medication therapy. This shows the potential of PGx implementation in a diverse population. Although research initiatives across the continent are advancing PGx knowledge, access to PGx testing remains largely confined to the private sector (Twesigomwe et al., 2025), limiting the benefits to patients who can afford testing. In the South African context, the principle of “ubuntu,” a shared humanity and interconnectedness, supports a vision for equitable access through PGx stewardship (Academy of Science of South Africa, 2018; Keuler et al., 2026).

Realizing the benefits of PGx testing for the broader population requires a healthcare workforce equipped with appropriate knowledge and competencies. Pharmacists, with their expertise in medication management, are recognized as core members of the PGx team (Fragala et al., 2022) and have been identified globally as leaders in PGx implementation, research, and education (Haga, 2023; Haidar et al., 2022). Pharmacists’ roles include communicating PGx information to patients and other health professionals (Alexander et al., 2014; Chart et al., 2021; Tuteja et al., 2013) and screening for potential adverse drug reactions and integrating PGx data into therapeutic decision-making (Alexander et al., 2014). Pharmacists working in PGx have acknowledged the complexity of this dynamic field and emphasize the need for foundational knowledge to interpret and apply PGx results effectively (Keuler et al., 2025). As the pharmacist’s scope of practice evolves to support personalized medicine (Alsaloumi et al., 2019), regulatory authorities and higher education institutions play a critical role in defining PGx and guiding PGx education.

The South Africa Pharmacy Council recognizes PGx as an important component of pharmacy education (South African Pharmacy Council, 2025a; South African Pharmacy Council, 2025b); however, there is no formal guidance on how PGx should be taught or incorporated into the curricula. This gap mirrors a global challenge. Pharmacogenomics education is neither standardized nor adequately integrated into health professionals’ training, posing a risk to patient safety (Kusljic and Luzum, 2024). Various authors have called for pharmacology curricula to embed PGx principles across graduate and continuing education (CE) for health professionals (Chang et al., 2019; Haga et al., 2016). As early as 2005, the International Society of Pharmacogenomics urged schools offering health professions education globally to incorporate PGx as a core component of pharmacology in pharmacy, physician and nursing education, and in continuing education for health professionals (Gurwitz et al., 2005).

With PGx becoming increasingly accessible to patients in South Africa, there is an urgent need to review and strengthen the curricula of health professions, particularly for pharmacy. A context-specific curriculum including PGx can prepare graduates who are prepared to meet the emerging healthcare needs in a diverse population and apply PGx in practice to improve patient care. This article synthesizes perspectives from international PGx experts to guide the evolution of pharmacy curricula in South Africa, ensuring that future pharmacists are prepared to meet the growing healthcare needs in PGx.

## 2 Methods

This study followed a qualitative study design inviting international PGx experts to participate in semi-structured interviews. A semi-structured interview schedule (Supplementary 1) was developed by the research team and refined following two pilot interviews with PGx specialists. Interview questions explored the role of the pharmacist in PGx, the knowledge and skills required for pharmacists to participate in PGx implementation, and recommended learning and teaching strategies for undergraduate, postgraduate pharmacy education, and CE pathways. Perspectives on community engagement were also explored.

Health professionals, pharmacists, doctors, nurses, genetic counselors, and laboratory personnel with PGx expertise were purposively sampled. Eligible participants met the following required criteria: affiliation with an academic institution, a minimum of 3–5 years of experience in PGx service delivery, and membership in a PGx-related professional organization. Having at least one pharmacogenomics-related publication was included as an additional criterion to demonstrate the experts’ experience in the field. Experts were invited to participate in a 1-h virtual interview via Google Meet. Various PGx organizations were approached to disseminate study information, and interested individuals completed a Google Form to provide consent for follow-up communication.

Interviews were audio-recorded and transcribed using Google Meet software. The researcher listened to the recording and re-read the transcriptions for accuracy. A detailed summary of the interview was shared with the experts for member checking. Transcriptions were analyzed using thematic analysis and an inductive approach in Atlas.ti. The first transcript informed the development of codes, and transcripts were re-read when new codes were developed until no new codes or themes emerged. The transcripts were analyzed by the researcher and supported by analysis from an independent research assistant (JeM) with experience in qualitative data analysis. The research assistant reviewed the study protocol and research questions to establish a foundation for data analysis. All transcripts were anonymized and verified by the researcher prior to being shared with the assistant. The researcher subsequently compared code reports and preliminary themes to identify areas of convergence and divergence, informing the development of the final thematic structure. Although the co-analysis contributed to refining themes and selecting supportive quotations, final adjudication was performed by the researcher following an iterative review process. The three overarching themes derived from this analysis are presented in this manuscript. An initial target of 15–21 experts was set; however, data saturation was reached after 20 interviews, as no new themes emerged.

The researcher is a PhD candidate from a developing country aiming to develop a PGx curriculum framework for pharmacy education in South Africa. As part of the PhD, she has observed PGx practices internationally, conducted an extensive review of global PGx education literature, and interviewed South African academics regarding PGx integration in pharmacy programs. These experiences may have influenced data interpretation. To enhance reflexivity and minimize bias, the researcher engaged in reflective note-taking using a guide adapted from Mack et al. (2005) after each interview and during transcription. Member checking was used to ensure that participants’ perspectives were accurately represented. Data were triangulated by comparing reflective notes, member checking summaries, and emergent themes.

## 3 Results

### 3.1 Demographics

Twenty PGx experts from 12 countries were interviewed over a period of 8 months. Most of the experts were pharmacists (13/20) and served on national and international PGx organizations, most commonly the Clinical Pharmacogenomics Implementation Consortium (CPIC) and the Pharmacogenomics Global Research Network (PGRN). Collectively, participants had substantial experience in PGx implementation, research, and education and were most commonly from the academic and hospital settings. Table 1 summarizes the demographics of the participating experts.

**TABLE 1:** Demographics of pharmacogenomics experts.

Number	Profession	Continent	Country	PGx experience (years)	Age (years)	Practice setting	Involvement in professional societies related to PGx/Involvement in PGx
1	Pharmacist	Africa	Egypt	6–10	41–50	Hospital, academic affiliation	CPIC member, PRGN (leadership), Standardizing laboratory practices in pharmacogenomics (STRIPE) member, African pharmacogenomics network (leadership) Leader at personalized medication management unit
2	Pharmacist	North America	USA	11–15	31–40	Academic, lead PGx pharmacist at hospital’s PGx clinic	CPIC member, PGRN (leadership), AACP PGx SIG (leadership), ACCP—pharmacokinetics, pharmacodynamics, PGx practice and research network sub-group (leadership); ISCC-PEG (leadership)
3	Pharmacist	West Asia (Middle East)	Jordan	21–25	51–60	Academic	ISSX (Science advisory committee, ISSX awards committee), PGRN (publication and implementation committee), CPIC, PharmVar, PharmGKB, ESPT
4	Pharmacist	North America	USA	11–15	31–40	Academic, PGx clinic, hospital (oncology)	PGRN (education committee, developing countries committee), CPIC, STRIPE (education committee)
5	Pharmacist	North America	USA	1–5	31–40	Academic, PGx implementation manager	PGRN, ClinPGx, CPIC, AACP—pharmacokinetics, pharmacodynamics and pharmacogenomics special interest group
6	Pharmacist	West Asia	Qatar	1–5	31–40	Hospital, PGx researcher	PGRN (implementation and global committee), CPIC Assistant director for the center for clinical precision medicine and genomics
7	Pharmacist	North America	USA	16–20	31–40	Academic, PGx clinic, hospital PGx residency coordinator	PGRN (clinical practice group), ClinPGx, CPIC, ACCP - PGx special interest group, PharmGKB
8	Pharmacist	North America	USA	11–15	41–50	Academic; PGx pharmacist PGx residency coordinator	ACCP - PK/PD/PGx practice research network (PRN); ASHP—writing group of pharmacist role in PGx, education; CPIC; PGRN
9	Medical doctor	Asia	India	1–5	31–40	Academic; provides TDM and PGx testing at hospital	CPIC, PGRN
10	Clinical pharmacologist	Africa	South Africa	6–10	41–50	Academic affiliation	European institution for technology and innovation in health Involved in medical legal cases which involved PGx Include PGx as part of answering daily clinical questions
11	Medical doctor	Africa	South Africa	21–25	>60	Academic, PGx researcher	Academy of Science of South Africa—human genetics and genomics in South Africa: Ethical, legal, and social implications Postgraduate research supervision in PGx, scientific PGx publications
12	Clinical Scientist	Africa	Zimbabwe	41–50	6–10	Academic, PGx researcher	African pharmacogenetic network, African society of human genetics
13	Physician	Europe	Spain	>30	>60	Hospital, academic, PGx researcher	CPIC, EMA PGx working group
14	Pharmacist	Australia	Australia	11–15	>60	Oncology PGx pharmacist, hospital, academic	Australian society of clinical pharmacology and toxicology—PGx SIG group, ESPT, PharmGKB, CPIC, PGRN
15	Research Scientist	Europe	Austria	11–15	41–50	PGx private laboratory, PGx researcher	CPIC, PGRN (scientific committee), ESPT
16	Pharmacist	Africa	Nigeria	1–5	51–60	Academic	APN, PGRN
17	Medical doctor	Europe	France	6–10	31–40	Academic, PGx clinic, hospital setting	French national pharmacogenetics network
18	Pharmacist	North America	USA	1–5	31–40	PGx clinic, community setting	PGRN
19	Pharmacist	Europe	France	11–15	41–50	Academic, oversee PGx clinics in hospital	French national pharmacogenetics network; IATDMCT - PGx committee; ESPT
20	Pharmacist	Europe	Netherlands	1–5	20–30	PGx clinic at hospital	PhD in PGx implementation, Masters in PGx implementation

AACP, American Association of Colleges of Pharmacy; ACCP, American College of Clinical Pharmacy; APN, African Pharmacogenomics Network; ASHP, American Society of Health System Pharmacists; EMA, European Medicines Agency; ESPT, European Society of Pharmacogenomics and Personalized Therapy; SIG, special interest group; IATDMCT, International Association of Therapeutic Drug Monitoring and Clinical Toxicology; ISCC-PEG, Inter society coordinating committee for practitioner education in genomics; ISSX, International Society of the Study of Xenobiotics, Leadership–participation in leadership: previous/current chair/president/founding member.

### 3.2 Identification of themes

Pharmacogenomics competency for pharmacists, approach to PGx education, and barriers to PGx education were identified as the three main themes with associated sub-themes. The quotations from experts (Es) supporting each theme are presented in separate tables (Tables 2–4). These themes are relevant across the continuum of pharmacy education, including graduate and postgraduate pharmacy education and CE.

**TABLE 2:** Theme 1: Pharmacogenomic competency for pharmacists.

Sub-theme	Expert
The role of the pharmacist in pharmacogenomics	
“There is no question for me that pharmacists should be well-instructed in the principles of PGx and actively involved in dispensing based on PGx profiles.”	11 (medical doctor)
“There are often misconceptions or misunderstanding on what is PGx and how it can be used. I think there is a large educational component that the pharmacist should be playing and providing to patients and the healthcare team, which for me includes nurses, providers, dentists, and your own colleagues, and then the general public needs to be better informed on PGx and what it can offer.”	4 (pharmacist)
“When I did this kind of the observation in South Africa … we found patients talked more to the pharmacist and pharmacy assistant regarding the medication. How should I take it? What could possibly go wrong? I know that sometimes the pharmacy and retail system can get quite busy, but pharmacists have the opportunity to talk more about PGx with the patient.”	10 (clinical pharmacologist)
“I think PGx education is probably the one that is applicable to every pharmacist regardless of any specialty training in PGx. I think PGx education is incredibly important not just for patients but also for providers, particularly in cases where there are clinical repercussions to a drug–gene interaction and dosing modification is recommended. I think the pharmacist’s role for education will be very important for helping providers understand how to dose medications based on PGx results. For patients, I think it is important that pharmacists are there to help explain, not just the basics of PGx, but also the benefits and limitations. Pharmacists are specially posed to answer that question based on the knowledge of metabolism, pharmacokinetics, and things like that….I look at research and clinical implementation as being quite similar.….I think on the research side of PGx, particularly where pharmacists fit in, is largely in the way that we distribute or implement PGx information….looking at what clinical and operational metrics you can gather by having a pharmacist-led PGx clinic and doing qualitative research as well on patient and provider experience.”	5 (pharmacist)
“I think this is our field to own as pharmacists. We have the skill set….I think, pharmacy right now has an opportunity to kind of step up and own this field. As a profession, we have an obligation to jump in and going from the clinical side from the research side, from the education side.” “We tend to use the term precision medicine pharmacists for genetic pharmacists, because I think there’s a little bit of a broader role for us than just in what we’ve traditionally defined as PGx … Demonstrating the value of a clinical pharmacist who has PGx experience specifically has been really successful for us. Well, it is still in the United States. There is a number of positions you have never really worked closely with, like a clinical pharmacist who is able to provide clinical-level recommendations and demonstrating that skill set and then weaving PGx into it as sort of a value add that has been a successful way for us to do this year.”	7 (pharmacist)
“We proposed to improve the vision of the lab to be a personalized medication management unit, and we had a reason why we did not call it a personalized medicine lab … So everywhere there will be that way of friction between pharmacy and physician. It is a border line. You do not know, is this a pharmacist or physician responsibility? We decided that there was only one question. If you can answer it, you will know if it is a pharmacist or physician. Is it medication related? It is a pharmacist….In 2016, we started to change our pharmacy lab into personalized medication management unit … If we are focusing on personalized medication management, we can get all things related to medication that can be personalized under the umbrella of the lab.”	1 (pharmacist)
“I think a futuristic role for the clinical pharmacist in the future is to play that role in implementing that [PGx], and I think that is going to be their duty to look after.”	10 (clinical pharmacologist)
“We [pharmacists] translate the genotypes to the phenotypes and in our daily clinical duties, we also authorize the PGx results … if there needs to be an intervention or not, and then it is most of the times communicated back to the doctor who will take action.”	20 (pharmacist)
Required knowledge and skills	
“I think it is obligatory to know everything that can help you to prevent ADRs. Pharmacists provide the drug and evaluate the prescription, and their main role is in drug safety by preventing ADRs.”	13 (physician)
Any pharmacist in my opinion in any country should know what PGx is and have the basic understanding of phase 1 and phase 2 enzymes and their role in ADME drug interactions. The extent of that education, I think, really is going to depend on what their actual authorities are in the healthcare system.”	15 (research scientist)
“I think they [pharmacists] need education. We are talking about basic knowledge on the way that we do the polymorphisms…. The most important part is how to read the reports that they get, what does it mean if they have these different stars alleles, and how to find the best recommendation depending on the guidelines, for example, CPIC, PharmGKB, how do they go around in these different websites to get the information that they need, and once they get the information, how to convey this information to the physicians or to their team and what kind of recommendations should they give. I think they have to learn that PGx is not the magic solution for everything. It is part of the solution to get more personalized medication or personalized pharmacy clinical practice.”	3 (pharmacist)
“Basic principle of pharmacology like pharmacokinetics and pharmacodynamics is going to be the foundation, the concept of drug interaction, and the basic concept of genetics.”	10 (clinical pharmacologist)
“I think a very in-depth understanding of drug metabolism, pharmacokinetics, and pharmacodynamics and then a marriage of the two how do variations in enzyme activity or even the absence of a particular enzyme, how does that impact on drug metabolism? What happens in different ethno-linguistic groups, and what would be the role of determining someone’s PGx profile?”	11 (medical doctor)
“Those skills would include literature evaluation, critically thinking about how research is being done in the area of PGx, how impactful the results of certain research studies that are done are, and then how to really pull together the different resources and make clinical decisions based on a variety of data. If CPIC has clinical recommendations that differ slightly from the Dutch PGx working group, how can you have those skills to critically think through which of those recommendations is going to be most applicable for your patient?”	5 (pharmacist)
“The other thing is the quality to work with multi-disciplinary teams; they should have knowledge on everyone’s role and how to work with them.”	6 (pharmacist)
“Having at least a basic, working understanding of laboratory testing in how these results are obtained in the laboratory; you do not need to be able to run those tests, but we need to be able to, you know, have informed discussion with the laboratorians. Understand the principles of others doing their work that you can understand where the limitations are different, and be able to have these conversations with laboratory colleagues.” “I think there is a common misconception among patients that this [PGx] is going to tell you exactly the right medication for you. How do we communicate the value but also the limitations of PGx testing so that patients have a good, informed understanding of what this test can or cannot tell? There is a lot more gray in PGx.”	7 (pharmacist)
“You need a very good communicator, a good researcher.”	1 (pharmacist)
Basic and advanced roles of pharmacists in pharmacogenomics	
“I use the analogy of infectious disease, as I think it is a good parallel to PGx, is that every single pharmacist has to know something about antimicrobial stewardship (AMS), right? Whether you are a retail pharmacist, whether you are hospital pharmacist, or whether you are a specialist, you have got to know the basics of AMS within different disease states or therapeutic areas. You have got to know a little bit more if you are an oncology pharmacist: you have really got to know the federal neutropenic guidelines. If you are a cardiology pharmacist, you have really got to know the endocarditis guidelines and how to treat endocarditis. But you still need an infectious disease pharmacist at the system level to help manage, you know, formulary and looking at antibiograms and all the different things that you have to do from a system management perspective to help things and then also be there for like the really difficult cases.”	7 (pharmacist)
“I go back and forth on phenoconversion and where that falls basic *versus* advanced.”	8 (pharmacist)
Basic roles	
“All pharmacists should have a basic understanding of genetics, the terminology, and the nomenclature because that is going to be important for interpreting the reports. I think all that boils down to all pharmacists should be able to utilize the information for patient-care management.”	4 (pharmacist)
“I think this is really important for hospital pharmacists and for pharmacists who plan to work in the industry field. It is maybe less the case for community pharmacists. I think or at least the content of the training has to be very different.”	19 (pharmacist)
“It would be phenomenal in the United States if, you know, our community pharmacist could be able to order PGx testing and interpret these.”	7 (pharmacist)
“Basic role for pharmacy is that they can do the awareness. This will be a great role; some hospitals do not have the lab inside the hospital, and they use a third-party lab. At least pharmacists can go to the physician, telling him, your patient is taking a high-risk medication. There is a PGx interaction here. Please order the test.”	1 (pharmacist)
Every pharmacist should be able to look at the results, go on ClinPGx, simple drug–gene pairs that have guidelines. They [pharmacists] should be make recommendations based off of that. I think every pharmacist should have this knowledge of what PGx is and what it is not. I see too many cases of “go to our PGx clinic and they are going to go through all your meds and tailor everything.” There is such a misconception among pharmacists even.”	8 (pharmacist)
“Two years from now, it should be a basic role when we see a pharmacist in a clinic counseling patients on their genomic information and on their drug–gene interactions.”	6 (pharmacist)
Advanced roles	
“It is really developing the structure, the groundwork for a site to be able to effectively practice or utilize that in their institution. That also includes analyzing metrics, being able to pull reports, develop metrics and quality control, and importantly, process improvement. I think the key is staying up-to-date on the field. I think it is a requirement for the advanced learner being able to continually be aware of new alleles coming up, new genotype or phenotype connection, new clinical implications, and so forth.”	4 (pharmacist)
“Taking into account not just a given phenotype with a particular dose-modification recommendation but really thinking about, if there are better options that can be used, are the other options associated with any of the genetics, and being aware of each of the genes that would be important in a clinical decision. Making sure that if a patient does have PGx results, for CYP2C19 by itself, do they also have results for CYP2D6, as that may affect one of the other medications that is potentially recommended or being used?”	5 (pharmacist)
“When it comes to advanced levels, this is where the next-generation sequencing comes into play where you take a particular set of patients, you take the blood, and you screen them for multiple possible genes and multiple possible SNPs, and you try to find out new variants for your particular group of patients. For example, South Africa, which is definitely going to be very different from people all over the world.”	9 (medical doctor)
*“*I think any clinical pharmacist can implement within their own practices at the level of patient care and that is where those core pharmacist competencies I would say are the baseline and a general understanding of the CPIC guidelines and navigating those resources. When it comes to more system-wide implementation and more larger-scale implementation, that is when those advanced skills that come out of residencies or fellowship programs can be helpful because implementation at that broader scale requires more leadership skills, collaboration with key stakeholders, leading the education of others and learning how to document these results in the electronic health record if that is available, and integrating clinical decision support and managing that service over time.”	2 (pharmacist)

**TABLE 3:** Theme 2: Approach to pharmacogenomics education.

Sub-theme	Expert
General approach to PGx education	
“I think one of the biggest hurdles in implementing PGx in clinical practice is education, and I am a firm believer that if you educate healthcare providers, pharmacists, physicians, and nurses, we can all agree that PGx is part of the solution in getting more personalized medicine*.”*	3 (pharmacist)
“I think this [PGx] is something that should be a core competency for every single pharmacist.”	7 (pharmacist)
*“*PGx is no more luxurious learning; it should be mandatory. Any pharmacist being recruited should have that type of education, and after recruitment, they should pursue mandatory courses; this will count toward their licensing. I feel for practicing pharmacists, they need to pursue education to keep their professional development. One element of these courses or conferences should be in PGx.”	9 (medical doctor)
“There has to be integration into the curriculum. It also needs to be standardized, especially if you are going to give the responsibility of managing PGx to a particular niche in the healthcare system. In order for that to be standardized, you have to put it into the curriculum.”	15 (research scientist)
“The paper is called PGx education strategies in the United States pharmacy school curricula, and it was a recent article review of different educational strategies, and the six include didactic lecture, personal genotyping or educational pharmacogenomic testing, simulation laboratory activities, inter-professional education, and practice-based activities such as a clinical rotation or combinational courses. Combining all different types of activities, they then advocate for a longitudinal curriculum design to make sure that this content is taught multiple times in many diverse formats over the course of their education at the school. I think that is a great approach. I think we do need a multifactorial approach. It is important to give the foundational content, but also that application piece, solving patient cases, doing simulation activities, getting out in practice and seeing it in real-world practice with a clinical rotation; I think are all great strategies. Some longitudinal design, multiple strategies like the ones mentioned, I think is ideal.”	2 (pharmacist)
“I get not being super prescriptive, but when we think about ACCP, it has the tiers tier one, two, and three for what to cover. I mean, they purposely call out that PGx is not a part of that. At minimum, then, I think we need to start thinking about if PGx in these cardiology states are tier one, then what are the PGx implications that go into that.”	8 (pharmacist)
“I think it is crucial to have PGx added into the curriculum. This can be in different ways; it can be integrated with other courses. I think it is important to at least get a lecture about what PGx is and get a workshop to get a feel about how to work with PGx results and give pharmacotherapeutic advice.”	20 (pharmacist)
“I think it should be an important part of the curriculum where the basics are taught at the beginning as part of your overall genetics or genomics education, but that it should be integral to every single organ system when it comes to talking about pharmacology and drug dispensing for each of those systems. I think it is the responsibility of the people who are running these courses to make sure that it is adequately covered at every stage along the way. I think it is the responsibility of each and every discipline in which there is a pharmacological option for treating a particular disease that each one of them then is responsible for teaching these principles along the way. The basics can be taught by somebody from basic science, preferably someone who is working in basic science who also has a medical background, but then it needs to be the responsibility of the people in every single discipline along the way because it is cross-cutting it cuts across all disciplines.”	11 (medical doctor)
Application-based PGx education	
*“*Flipping classroom, a case-based scenario is going to be quite important.”	10 (clinical pharmacologist)
“Focusing on specific therapeutic areas with the drug–gene pairs and tailoring that depending on your audience of pharmacists, their patient population, and what drugs they are commonly using and then focusing on those that have relevant CPIC guidelines so they can see the relevance to their practice. Just to add on with the case studies part, I think it should be the individuals navigating PGx resources, having practice going to the new ClinPGx site.”	2 (pharmacist)
“I think the part that really gets the home point with the students is to have very well-written clinical scenarios that demonstrate where PGx can be and often is a very important consideration in general clinical decision-making. I Think that what I have seen work the best in the past and what I think worked really well for me is going over some of the basics of PGx in the very first part of the course and then maybe learning how to apply it in different scenarios. That is where you could have trick questions related to prodrugs or clinical scenarios that really require the learner to think past just here is what CPIC says about this drug–gene interaction.”	5 (pharmacist)
“I think a didactic experience is important. I think this is not just unique to low- and middle-income countries; it is the ability to then offer hands-on experience or workshops that would be helpful in solidifying concepts.”	4 (pharmacist)
Story-telling	
“What is much more helpful is if you tell a story that is interesting and bring some of the details of the whole field, but you can talk about slow or ultra-rapid metabolizers. You can talk about distribution across the world, but then you need to link it to something that is practical and provide a whole bunch of websites where people can go and look up specific things; but they need to understand the principles. I think it is really important to talk about practical problems. What does CYP 2D6 null mean? What does it mean if you need to go on to an anti-depressant? What does an adverse drug reaction look like, or what it would mean if 50% of the population does not respond to the standard dose of a drug because we have not adequately looked at how that drug is metabolized and what the genetics are in that particular population. From that to issues around cost, what is the cost of the adverse drug reactions due to hospital admission? And how could we better spend that money if we were not spending it on treating people that are sick because of the medicines we give them?”	11 (medical doctor)
Pharmacogenomics topics to be included in pharmacy education	
“I would expect them [pharmacists] to be exposed to basic genetics concept, basic pharmacology concepts, and pharmacokinetics—that is going to build your foundation. I think there is that sort of PGx concepts—haplotype, alleles, activity scores, metabolizer status, different types of genetic variation, copy number variation, and things like that. That is what I would expect to be part of the first third of a PGx course, and then the last 2/3 of a PGx course should be focused on therapeutics. So how do we apply this in practice? There is probably value in people getting exposed to some higher level concepts; e.g., is this regulated and reimbursement, which would be country specific, but understanding the economics and role of regulatory bodies in this information is really, really critical, at least in the US that is a super-hot topic right now of what is the role of the FDA?”	7 (pharmacist)
“We teach students how to look at a continuous glucose monitoring report and read it. So, why would we not teach them how to look at a PGx report? I think we train them in counseling and writing notes. We train them in how to talk to clinicians and patients. So, we need to train them to do that with a PGx aspect. I think we are teaching these disease states already. Why would not we just add in the valuable PGx information that goes along with it, especially if it means a dose change or a med change or monitoring? I think obviously you have to cover the basics, but I feel really strongly on those higher levels of bloom taxonomy of learning and focusing on the application of it and less of the memorization because things are changing so quickly.”	8 (pharmacist)
“I think by making sure that at least basic foundation of genetics can be covered at the BPharm one or two by the time you are going to go for the BPharm three and BPharm four, you can establish clinical PGx when they make more sense about bringing the basic foundation to the clinical foundation when it is relevant. So, reverse education I think is going to be the tool, which involves more clinical cases, let the class be interested about what is happening and then get them involved; then, you can introduce the concept of PGx as a lifesaving tool that could prevent this even from happening, and you can even walk the extra mile by talking about the medical legality behind it.”	10 (clinical pharmacologist)
“I think all the issues related to genetics and genomics, whether it be disclosure of personal information, reidentification from whole-genome sequences, related to intellectual property or genetic counseling, these apply across the board, and I do not think what one can single out PGx relative to anything else. I think the principles apply across all domains, so I would not single out PGx as being particularly important. I think all areas are important, and I think the topic of the ELSI of genetics and genomics should be taught as a separate topic, but that should also include but not be limited to PGx.”	11 (medical doctor)
Activities for learning and assessment	
“Infographics as part of their assessment”	3 (pharmacist)
“I had students help me design a PGx clinic. I asked them, “what does the clinic look like?” That is one example of a research project that I had students do, and that is more on the clinical side of it, and they did it with good success. We now have a good blueprint for a clinic that we are ready to launch. Another example is developing a survey. I ask students to develop a survey to ask our doctors, do they want PGx?”	4 (pharmacist)
“A lot of our assessments in our courses are multiple-choice question exams. I think that works well for foundational knowledge and concepts. I do think it is important, if possible, to work in those skills-based assessments in terms of their ability to work up a patient case, navigating resources to answer PGx-related questions.”	2 (pharmacist)
“I think OSCE would be number one…. I cannot assess something related to the patient without having a real case or a role-play model. If there is possibility to make a shadow training in a hospital in PGx, this will be more than perfect.”	1 (pharmacist)
“Virtual reality right, so why not use case scenarios with the virtuality. You give a case with the patients who are coming for counseling.”	6 (pharmacist)
“For me, the best way to get anyone to really understand what PGx can do, and this is a lay person all the way to a medical physician all the way to someone who is a pharmacogeneticist is to do their analysis, give them their PGx analysis and say, “this is you.” You are a poor metabolizer 2D6. For the rest of your life, you can tailor any drug that is metabolized primarily by that gene….I would get a set of dummy reports and have a workshop on it where you have the same patient analyzed by different entities with different content. See how the recommendations differ and see what how you manage this.”	15 (research scientist)
“When we work on personal genotyping, for the assessment, we ask the student to identify using the knowledge resource what should be tested and what kind of variant should be tested. We ask them to work using PharmGKB, pharmvar to understand the concept of these databases, and to be sure that they can use the databases by themselves. This is a good thing to have kind of exercise using the databases because this is what we do when we use PGx. We are looking for what recommendation, what test, and what variant.”	19 (pharmacist)
“Experiential learning, I think, is the ultimate, getting them in the real world and interacting with real patients and real patients’ results and seeing how it is applied to care. I think that is really very high-impact learning activity.”	2 (pharmacist)
“We throw our students [APPE] right into the mix that they are working up patients with us. We will have patients who have received PGx testing that we are working through, and they will get assigned a patient. They will be working up a patient, a chart note, and a recommendation for that patient; we will then review it, we will talk about it. We have our weekly PGx clinic where we see patients in person, and it is a 4-week rotation. The first two weeks, they are just kind of observing, and maybe doing some of the documentation on the back end. The expectation is that by the end of the rotation, they will be able to lead a session and educate the patient. We have them do the pre-test, not so much the post-test, but kind of a pre-test session where they are kind of educating the patient on the benefits and limitations of testing.”	7 (pharmacist)
“We have the student shadow our clinical PGx-trained pharmacist and basically make sure that any kind of PGx inquiries that we have from providers or patients, which do come up frequently, are being answered with help from the student. This would help solidify some of the PGx concepts in real pragmatic ways as we do get quite a few questions. We additionally do see patients for PGx testing. So that could be a referral from a provider and then basically going through the entire process of meeting with the patient describing PGx in general, its benefits, its limitations, and what is involved in the testing, and then interpreting the results and providing an answer to the referring provider’s inquiry, and then providing the information that comes from the report that was generated as a whole to the patient. We really involve our students in every step of those types of processes because it is pragmatic. It is what we do in clinical practice, but it really allows students to go through each piece of the experience that is involved in providing PGx clinical care.”	5 (pharmacist)
“In our curriculum, there are 6-week clinical rotations, and I offer one in clinical PGx, and students are involved with my PGx clinic. They are looking at PGx test results; they are interpreting it and translating genotype into phenotype; they are going to the guidelines to come up with specific medication recommendations and take into context the patient’s other conditions and other medications. Then they are involved with the patient visit and observing. And sometimes, the patients may engage them with specific questions. We always debrief after patient visits and after the clinic with the whole team, asking the students about what they have observed and their impressions about what happened, any questions they have. They are kind of integrated into that clinic process. They are also involved with other activities which include discussions about CPIC guidelines. They need to review the guidelines and come ready to discuss the drug–gene relationship and the clinical recommendations. I have them attend CPIC calls so they see what is happening globally, what are the upcoming guidelines, or sometimes as presentations from individuals who are implementing; we also do journal club activities, we are analyzing PGx-related literature, and then other projects I may assign to them really depend on the specific projects that I am working on and what the needs are of the clinic. For instance, in the past, if we needed a patient education hand-out, they would be working on that. I try to engage them in the work that I am doing as a PGx expert involved with research and education and patient care, and that is the inspiration for the projects that they are assigned.”	2 (pharmacist)
“During internship, we have clinical duty of the pharmacist, they [pharmacist] get the genotype results, and they need to interpret them for the patient and what it means. That part is where we learn a lot during our internship as well as talking with the laboratory pharmacist who is responsible for the PGx testing on how they have implemented it in their daily clinical practice. I think that is the most important aspects during the internship.”	20 (pharmacist)
Community engagement in pharmacogenomics	
“Personalized medicine opens the door for patients to do their part in participatory medicine. When he [health professional] has a PGx test, he tells the patient first you have a certain mutation, I have to change the medication to a more expensive medication to avoid the side effect, and at that time, the decision is a participatory one where the patient can participate in this decision. I think PGx and personalized medicine open the door for patients to participate in the decision.” “I think this will be very helpful for them as students so that when they graduate they can understand the importance of reaching the patient with the information.”	1 (pharmacist)
“I think so, but I would have to be careful on what on how the student is communicating it. The reason why I say this very hesitantly, is we have pharmacists who do not know the whole aspect of PGx and would make erroneous comments about it as a pharmacist. Not to their fault. It is a really nuanced field, right? And so there is a lot of unique aspects that not everyone knows. I would say I am a little bit okay with a resident talking to a patient on what to do, but maybe not so much to the general public. That interaction to me is very different. Me interacting with a patient *versus* me interacting with the general public.”	4 (pharmacist)
“I absolutely think it can be very helpful, from the undergraduate experience, maybe thinking about it in a little more general term. So, having an undergraduate describe to community or patients. A broad overview of PGx.”	5 (pharmacist)
“I am worried about the ethics about that. I mean, I do not know I might not be incorrect here, but I mean, you understand because this is the topic that touches the line of ethics talking about genetic testing, but talking about awareness is very important.”	10 (clinical pharmacologist)
“I think it is much more meaningful if you were to focus on the healthcare provider and the community because the one without the other certainly not having a healthcare provider who can respond would be meaningless and, as I say, may lead to greater frustration.”	11 (medical doctor)
“I do think this could be a really interesting area for students to get involved with. I think this can benefit both the students and the community. We know there is a need for patient education in PGx, and so maybe students can do something on social media or YouTube videos to educate patients about PGx and where to get reliable PGx information or even giving little presentations to members of the local community in different organizations within the community that might be interested in those sorts of talks to help get the word out to individuals and patients about PGx.”	2 (pharmacist)
“Infographics for sure to be sent to patients. Those infographics can be utilized to educate and provide awareness to the general public about PGx because patients have the right, and also the community.”	6 (pharmacist)
“Two students have done work in community engagement of pharmacists in my country in terms of PGx.”	12 (clinical scientist)
Integration of pharmacogenomics across the continuum of pharmacy education	
Graduate pharmacy education	
“I think first, it should not be an elective, it should be very a mandatory course.”	6 (pharmacist)
“The people who have that standalone course are better able to handle the content that we teach them on rotation. Because it has been presented to them in more of a formal way, *versus* the people who just got a lecture here and there and have it kind of scattered across the curriculum.”	7 (pharmacist)
*“*I have always advocated for integrated. In my program, I do an introductory lecture for the students, and then it is kind of integrated throughout the therapeutics courses. I provide guidance to those faculty teaching in those areas; e,g, here are the drug–gene pairs that are important and the basics of the recommendations, and then they can integrate that as they are teaching the treatment of depression say right and now they are integrating the importance of CYP2D6 and CYP2C19 and it is just part of the whole picture for the patient, so you do not want students to see PGx as kind of something over there as separate from the typical care and really only for specialists. You want to have them see it as this is just part of how we evaluate a patient, just like any other clinical variable. I think with the integrated approach, another benefit is that it helps reinforce the material over time, which can help increase learning and retention of that information.”	2 (pharmacist)
Allocated credits for PGx	
“I think it is a difficult question. I do not even know if I could tell you with my own program right now because of the integrated format. It is hard to quantify that. It is more of we have identified these are the drug–gene pairs that should be integrated in some way, and we kind of leave it up to the individual instructors to do that as they will at this point. Right now, I have maybe 2 hours of foundational content in the beginning of their therapeutic sequence. It gets integrated throughout many different courses that last the next couple of years, and then I kind of come back toward the end of their didactic curriculum to give another 2-h lecture on more advanced topics and implementation and integrating into practice. That is just what we have time and space for in our curriculum. I think it is always a struggle to be adding content to the curriculum because it is already so full, so I think that is probably a minimum of what should be done. I do know there are other programs that have dedicated courses, but again, I think it is ideal to be integrated. I think it increases learning and retention over time. It might be best to go based on objectives and what can fit.”	2 (pharmacist)
“PGx should not be more than 5% from the whole credit. This is not to inferiorize PGx. It is hard, the weight of it, because as I said, it is part of another foundation. Pharmacology as a big umbrella is a really important one. PK PD is the second umbrella, and it falls there with the drug–drug interactions. So if you start with 5% and as the new sciences and new research showing the fact that this is to be saving, if you see the future when there is a digital prescription that actually considers adding PGx setting of the patient to the prescription, then we can talk about revising that kind of percentage.”	10 (clinical pharmacologist)
“For PGx, I do 10 h, and that is the 10 that I teach ”	12 (clinical scientist)
“So for 10 weeks, that will be 30 h”	16 (pharmacist)
“Maybe 20 h”	18 (pharmacist)
“I would say 14 h–28 h”	20 (pharmacist)
Postgraduate pharmacy education	
“You introduce this [PGx] in the undergraduate level and then you start a specialized fellowship in PGx where this person who has had this basic knowledge in the undergraduate level goes on to specialize in PGx where you do your own GWAS study. You get your PhD published, and then you can go and work as a specialized pharmacist who has specialized or done a PhD in PGx; that is how I feel this should go.”	9 (medical doctor)
*“*When we get into residency, we are focused on applying those skills and practice. In our program, the first 2 months are heavily didactic, where we are getting people up to speed. We do a lot of topic discussions; we do a lot of education. They go through every single CPIC guideline and summarize it, and so they are doing a lot of core knowledge work, and then at the same time, they are working up patients and applying those skills in practice. Then they are going out into different rotations outside of the precision medicine departments. They are going out on a cardiology rotation, or a primary care rotation, or an oncology rotation that they can be the PGx expert within that disease. That is where I am hoping that they are getting those skills. They do not need to know everything that an oncology pharmacist needs to know. They need to know enough of the therapy and enough of like how things work in oncology that they can interface in the future within oncology pharmacists in that area and be able to kind of speak knowledgably about how things work in that setting. I think, in residency, it is more of those soft skills of how do you take this knowledge that you gained and put it into practice and work with people? How do you start a business? You set a project plan, and timelines, and be able to execute on our project; that is what we are working on in residency.”	7 (pharmacist)
“For our residency, we do mostly longitudinal and not block modules. It is not like you spend a month in this and then you are done. That allows us to ramp up or down, and we make sure we cover the requirements, but again, if they are more interested in one area, then we can wrap that up and just make sure we cover the basics of something but not spend a lot more time on it … We have our resident doing a lot of our chart reviews. They are seeing a good chunk of patients, but our first month is orientation. We do a ton of reading of the guidelines, going through some modules that we previously prepared. Then, we are heavy on the topic discussions right away. We do a lot of topic discussions, and we continue topic discussions throughout the year, but then we generally transition it eventually, and then the resident is leading the topic discussion with the APPEs or the PGY1s. Didactically, we spend a lot of time in the guidelines and doing topic discussions. They have to do journal club once a month, and we alternate so that it might be an admin leadership journal club or might be clinical, so they get that experience of not just presenting on clinical articles. For our CDS journal club, we invite the IT team, and they come and they ask questions. So, we try and do multidisciplinary.”	8 (pharmacist)
Continuing education	
“We do need to make sure the pharmacists understand why it is important, thinking about how it is relevant to their practices, and creating educational opportunities that can fit into their busy schedules.” “I have seen examples of how the offering of educational testing can also be helpful for the practicing pharmacist because it gets them engaged and thinking wow, this is really neat. So that can be a helpful tool, and then also incorporating real-world case studies as much as possible. This is actual patients that an expert or someone who is a clinician or whatever has seen that is relevant to those pharmacist’s practice areas and show how PGx is important and how to apply it to patient care using these real-world case studies. So, I think that can often speak volumes when they see wow, these are real patients that really benefited from this, and then they can think about how that may apply to their own patients and their own practices.”	2 (pharmacist)
“Perhaps reviewing a given area of medicine and how PGx relates and then providing information that may be new even if the student or practicing pharmacist learned it as part of their studies. Introducing something new and then still including that clinical component to kind of bring it all home. If you were doing a continuing education, have overview basics of PGx and cardiology, we have new guidelines; for example, for this medication, here is new information, and then, at the end of the day, you are bringing it together with at least a couple of clinical cases to really utilize that information in ways that pharmacists are likely to encounter it in everyday practice.”	5 (pharmacist)

**TABLE 4:** Theme 3: Barriers to pharmacogenomics education.

Subtheme	Expert
Need for standardization of PGx education standards	
“It [PGx] needs to be in the curriculum. There are no real standards on what to cover or how in depth. I think there needs to be more guidance. I do not want more prescriptive guidance on all the things that we need to cover, but I think just like a one-line PGx is too broad and there is too much inconsistency and there are too many people saying that they cover it but not in a way that is valuable to students.”	8 (pharmacist)
Limited capacity among faculty	
“I think, for higher education institutions, the biggest thing would be if we train our faculty, the professors and everyone, that would definitely help because if they know about something, they can pass it on to their students.”	18 (pharmacist)
“I guess what is happening here at the university for me anyway, I am the only expert in the area, and so I do all of the teaching when it comes to this area. My recommendation is to find someone who knows what they are doing, knows what they are talking about.”	4 (pharmacist)
Overloaded curriculum	
*“*The curriculum is chock-and-block full. So the question is how does one effectively integrate PGx? I think it has to be integrated, but although it is our favorite topic and we would like to see it given much more attention realistically and practically speaking, there is a lot of stuff that needs to be covered. So, one way of doing it would be to integrate it into the different modules in training, whether it be in medicine or pharmacy. I teach the cardiovascular system all the way from anatomy through to pathology, clinical applications, etc., which includes the pharmacology. When we talk about particular medicines that are used to treat patients for cardiovascular disorders, we should know which of those would have practical PGx implications, and we should do that all, not just for cardiovascular disease but for every discipline along the way. So it is not every single drug, as we know there are some for which there are adequate data and it has been adequately shown that PGx intervention makes a difference, but it should be introduced not as a once-off one or two lecture coverage of the topic, for example, in the first 3 years, but it should be integrated at every point along the way as an integral part of prescribing, whether that is in medicine or in pharmacy.”	11 (medical doctor)
Readiness and practical implementation requirements for pharmacogenomics	
“We need to take one step back and understand what is the *status quo* of PGx in the country. The reason why I am saying that because that is going to be exactly what kind of message I have to convey to the pharmacist, is it available? The answer is yes. Which medication/category is available for if the answer yes, is how much is it going to cost and do the medical aid cover that? Because backed by these information, the pharmacist can even recommend this kind of tools for the next treatment, but I think we need to have a consensus regarding that and knowing it is one fact but what to do with the information to me is the most important crucial fact. I know about PGx. I know it does exist. It is going to help patients, but the question is which lab? Can I send the patient to lancet to do that? I sent a doctor to contact pathcare and said I want to do genetic testing for my patient, and what is the protocol? Can anyone do it? Does it need a sub-specialist to do that, and can medical aid pay for that? I think, if we provide the pharmacy with this kind of information, like listen this tool is available in South Africa, is recognized there is certain level can be done that and what you can do, for example, we can open a dialog between them and doctors. You can contact the doctor, listen, as a pharmacist, we think this medication is not doing its job because one two three, and there is a recommendation from our consultant and SAPC or PSSA to say send the patient for the PGx, and here is the recommended laboratory services who can do this testing and can feed you back by understanding the feasibility access and legality of that. I think it is going to be fantastic to make sure that the pharmacist can do the job properly.”	10 (clinical pharmacologist)
Access to facilities that offer PGx testing/services	
“I would say lab work definitely helps and the case studies, because I come from a low-resource environment. The resources that I get are also limited … What I would really like the students to observe is a real case, not the ones we give them on the theoretical ones, a real in-house clinical case where I wish we were exposed to clinicians that are able to pick up PGx testing cases. I wish the students would be able to see how the clinicians request for these tests, how these tests are actually done, how this information comes back to the patient, and also observe the counseling that is given to the patient when these results come and those change. I wish we could do that.”	12 (clinical scientist)
“Ideally that we have a lab and I have perhaps a hospital that I can send the student to get trained on the ground by pharmacists or clinicians who are involved in PGx, whether in research or whether in clinical settings. Unfortunately, I do not have that because simply the number of people applying for PGx is very small and it is not a common practice to find somebody who actually practice in PGx.”	16 (pharmacist)
*“*I think it is hard to think about experiential learning just because so many places are not implementing it. I have my students who are on rotation with me; they are reviewing the results, and they are writing the note in the chart. They are helping me with quality improvement projects, and they are seeing the patients with me in clinic.”	8 (pharmacist)
“Because we have so many students, sometimes it is hard to give them hands-on experience. I think learning by just looking and watching people doing this and case-based studies, but for postgraduate students, the proposed idea is to get them into the lab and to give them actual samples to work on and for them to know exactly what kind of problems they might face and how to read the reports after that. This is in the postgraduate I think in terms of the learning should be more on experience than when we talk about the undergraduate students. Now, one of the things that we can do because students are exposed to a lot of cases from the hospital is they can look into hospital records. They can go through the reports of cases of actual patients, and they can see if they can recognize certain drugs that might benefit from PGx testing other than the obvious ones. For example, we are talking about warfarin and clopidogrel, oncology drugs. Maybe we are talking more about certain types of statins; for example, why certain patients will have a lot of side effects with statins? When they switched to another type of statin, did they have the same type of problems or not? If this is one of the experiences, that they can learn by looking at the actual cases and pick up what the red flags are in terms of drugs and if PGx can be part of the solution for these individuals.”	3 (pharmacist)

#### 3.2.1 Theme 1: Pharmacogenomic competency for pharmacists

This theme explores PGx competency for pharmacists. The sub-themes identified include: 1) The role of the pharmacist in PGx, 2) required knowledge and skills to practice PGx, and 3) basic and advanced roles of pharmacists in PGx. Theme 1 and the subsequent sub-themes are summarized in Table 2.

##### 3.2.1.1 The role of the pharmacist in pharmacogenomics

Experts consistently emphasized that pharmacists occupy a central position within inter-professional PGx teams, contributing across education, research, and clinical implementation. Among these domains, education emerged as the most critical and universally applicable role, with experts noting that all pharmacists, regardless of practice setting or specialization, require sufficient competence to educate health professionals, students, patients, and the public on PGx (E4 and E10). Pharmacists’ deep understanding of pharmacotherapy enables them to address misconceptions, clarify drug–gene interactions, and support safe use of medication through PGx testing and medication management (E4 and E20).

Several experts argued that the pharmacy profession should assume leadership in precision medicine, particularly in research, education, and implementation (E7). Clinical pharmacists were identified as key partners to collaborate with prescribers, with the potential to lead PGx implementation efforts in South Africa (E10). Across interviews, the importance of close collaboration between pharmacists and doctors was repeatedly highlighted as essential for effective PGx integration into healthcare. Expert 5 highlighted the importance of research and implementation taking place in parallel to improve practice.

##### 3.2.1.2 Required knowledge and skills for pharmacists to work in pharmacogenomics practices

Experts agreed that pharmacists require a strong basis in foundational genetics and genomics, along with pharmacokinetics, pharmacodynamics, and pharmacovigilance, to competently interpret and apply PGx information in practice (E3, E10, E11, E13, and E15). The ability to navigate PGx resources and guidelines was identified as essential for making evidence-based recommendations for PGx testing results (E5). Given the evolving nature of PGx, pharmacists must also be able to critically evaluate discrepancies between guidelines and exercise professional judgment in areas of uncertainty (E7).

In addition to technical knowledge, experts emphasized the need for strong communication and interpersonal skills to support inter-professional collaboration and to convey PGx information to both health providers and patients at the appropriate level (E1). Experts agreed that these competencies enable pharmacists to facilitate informed decision-making and enhance the clinical utility of PGx testing.

##### 3.2.1.3 Basic and advanced roles of pharmacists in pharmacogenomics

Experts distinguished between basic and advanced PGx roles. There was consensus that all pharmacists should possess basic PGx competence, including foundational genetics knowledge, the ability to interpret PGx reports, navigate freely available evidence-based PGx resources, and provide PGx counseling (E4 and E8). With this baseline competence, pharmacists can contribute to raising awareness of PGx and supporting safe use of medication (E1). Expert 8 debated whether more complex concepts, such as phenoconversion, should be considered basic or advanced knowledge, reflecting ongoing uncertainty in defining the competency thresholds.

Pharmacists with advanced training, such as postgraduate qualifications or specialized clinical experience, were viewed as well-positioned to lead PGx implementation within institutions, conduct PGx-related research, and manage complex cases involving multiple gene–drug interactions (E2, E4, E5, and E9). These pharmacists may also serve as consultants within inter-professional teams, providing expert input on precision medicine strategies (E1, E7, and E10).

While experts acknowledged the value of pharmacists across settings, some noted that the extent of involvement may vary. Community pharmacists, for example, may have fewer opportunities for direct PGx engagement (E19). However, others argued that community pharmacists are uniquely placed to identify patients who may benefit from testing and could even initiate PGx-testing in appropriate contexts (E7 and E10).

#### 3.2.2 Theme 2: Approach to pharmacogenomics education

This theme describes a general approach to PGx education, education topics, activities for learning and assessment, community engagement, and recommendations for the level of pharmacy education. The themes and sub-themes are summarized in Table 3.

##### 3.2.2.1 General approach to pharmacogenomics education

Experts consistently identified PGx education as a foundational pillar for successful PGx implementation and for preparing pharmacists to participate meaningfully in precision medicine (E3, E7, and E9). Pharmacogenomics was described as an essential competency for all pharmacists, and experts (E8 and E11) recommended a tiered learning model that differentiates between foundational and advanced PGx competencies across the training continuum.

Experts supported the integration of the PGx content into existing modules and emphasized that every discipline within the pharmacy curriculum has a role in embedding PGx concepts where relevant (E20). This integrated approach was viewed as more sustainable than isolated PGx teaching and better aligned with how pharmacists encounter PGx information in practice (E2 and E11).

A strong emphasis was placed on context-specific, application-based learning, particularly focusing on actionable gene–drug pairs and the ability to navigate evidence-based PGx guidelines and resources (E2, E4, E5, and E10). Experts advocated for a longitudinal learning experience that revisits PGx concepts across the curriculum to support progressive competency development (E2).

To enhance retention and promote clinical reasoning, experts recommended the use of storytelling (E11) and case-based scenarios (E2 and E10). These methods were seen as effective for helping students internalize complex concepts, apply PGx principles to real-world situations, and appreciate the clinical implications of PGx-guided decision-making. Experts also stressed the importance of discussing the clinical and ethical implications of PGx concepts to ensure that students develop both technical competence and professional judgment (E11).

##### 3.2.2.2 Pharmacogenomics topics to be included in pharmacy education

Experts emphasized that pharmacists require foundational knowledge in genetics and pharmacology as the basis for competent PGx practice (E7). Beyond theoretical knowledge, students should also understand the practical aspects of PGx, including regulatory requirements, reimbursement considerations, and how PGx testing is operationalized in real-world healthcare settings (E7).

To support deeper learning, experts highlighted the need to incorporate higher-order thinking through application-based teaching strategies that move beyond memorization to interpretation, evaluation, and clinical decision-making (E8 and E10).

Ethical, legal, and social implications (ELSI) were also identified as important components of PGx education. While one expert suggested that ELSI topics could be integrated within broader genetics teaching (E11), there was consensus that students must be equipped to recognize and address the ethical and societal considerations inherent in PGx testing and precision medicine.

##### 3.2.2.3 Activities for learning and assessment of pharmacogenomics

Experts emphasized that PGx education should prioritize practical and clinically relevant learning activities that prepare pharmacists to translate knowledge into real-world practice. Oral and written examinations were identified as suitable assessment methods (E2), while personal genotyping was highlighted as both a powerful learning tool and an assessment strategy that deepens students’ engagement with PGx concepts (E15 and E19).

Experiential learning emerged as a particularly valuable component of PGx education (E2 and E20). Experts described opportunities for students to observe and participate in real-world PGx practice, including patient counseling, interpreting PGx test results, translating genotype to phenotype, and even attending monthly meeting calls from CPIC (E2, E5, and E7). Several experts currently integrate students into routine PGx activities to provide hands-on exposure to education, research, and implementation.

However, experts acknowledged that access to real-world PGx practice varies by location and institutional capacity. In settings where experiential opportunities/resources are limited, simulation-based approaches, such as objective structured clinical examination (OSCEs) and structured application-based activities, were recommended as effective alternatives to ensure that students still develop practical PGx competencies (E1, E4, and E6).

##### 3.2.2.4 Community engagement in pharmacogenomics

Experts expressed mixed views on the role of community engagement in PGx education for pharmacy education. While there was broad agreement that patient and public education is an important component of PGx practice, several experts noted that the students’ level of knowledge and experience should determine the extent of their involvement in community-facing activities (E4 and E5). Experts agreed that pharmacy students can contribute meaningfully to general PGx awareness and education for patients and the public, provided that activities are aligned with their competency level (E1, E6, and E10).

In resource-limited settings, community engagement has already been incorporated into PGx training. For example, Expert 12 supervised a student research project focused specifically on PGx-related community engagement, demonstrating its feasibility even in constrained environments. Experts also highlighted that health professionals play a key role in empowering patients to participate in shared decision-making through personalized medicine approaches (E1).

Finally, experts emphasized that health professional education should occur in parallel with community education to ensure consistent messaging and to strengthen the overall ecosystem of PGx literacy (E11).

##### 3.2.2.5 Integration of pharmacogenomics across the continuum of pharmacy education

###### 3.2.2.5.1 Graduate pharmacy education

Most experts recommended that PGx be integrated into the existing pharmacy curriculum as a core component rather than delivered as a standalone module (E6). Although some experts viewed a dedicated PGx module as ideal (E7), the broader consensus was that integration is more feasible and effective, as it allows students to encounter PGx concepts repeatedly across different contexts (E2). Experts supported a tiered approach in which a foundational PGx course is introduced early in the program, followed by integration of PGx content into pharmacology and therapeutics modules to create a longitudinal learning experience (E10 and E11).

Experts acknowledged that allocating specific credits or hours to PGx education is challenging because PGx is inherently embedded within multiple therapeutic areas (E2). One proposed solution was to assign a percentage of module time to PGx content or to align teaching with clearly defined PGx learning objectives (E10). Across institutions represented in the study, the number of hours currently dedicated to PGx ranged widely at 2 h–30 h, reflecting significant variability in curricular emphasis and institutional capacity (E12, E16, E18, and E20).

###### 3.2.2.5.2 Postgraduate pharmacy education

Experts identified postgraduate training as a pathway for developing advanced PGx expertise. A PhD was viewed as a key opportunity for pharmacists to specialize in PGx, particularly for those interested in contributing to research, evidence generation, and academic leadership in the field (E9). In addition, experts highlighted the value of a PGx-focused residency program as a unique avenue for acquiring advanced, practice-based skills through intensive experiential learning (E7 and E8). Residencies are seen as critical for preparing pharmacists to lead PGx implementation efforts, manage complex clinical cases, and support the integration of precision medicine within healthcare systems.

###### 3.2.2.5.3 Continuing education for practicing pharmacists

Experts agreed that pharmacists should understand the clinical relevance and healthcare impact of PGx to participate meaningfully in precision medicine practices (E2). Experts emphasized that PGx education should be readily accessible, clinically focused, and offered through ongoing continuing professional development to ensure that practicing pharmacists remain up-to-date with evolving evidence (E5).

One strategy to enhance engagement is the use of personal genotyping, which experts described as a powerful experiential learning tool that allows pharmacists to interact directly with PGx data and appreciate its clinical implications (E2). Incorporating real-life clinical scenarios was recommended to strengthen application-based learning and support the development of practical decision-making skills. The latter is feasible in resource-constrained settings.

#### 3.2.3 Theme 3: Barriers to pharmacogenomics education

The third theme focused on barriers to PGx education (Table 4). The need for standardization of PGx education standards, limited capacity among faculty, overloaded curriculum, readiness and practical implementation requirements for PGx, and access to facilities that offer PGx testing/services were identified as sub-themes.

Experts identified several barriers to strengthening PGx education for pharmacists. A key challenge is the lack of structured guidance and standards for PGx education, which limits consistency in teaching and makes it difficult to ensure that graduates achieve the required competencies (E8). Incorporating PGx into already dense pharmacy curricula requires a pragmatic and balanced approach to avoid further curriculum overload, particularly when aligning with global pharmacy education standards (E11).

Experts noted that many higher education institutions lack staff with specialized PGx expertise, making it difficult to deliver high-quality, practice-relevant PGx education (E4 and E18). Understanding the current landscape of PGx implementation and testing within a country was viewed as essential for providing context-specific education that prepares pharmacists for the realities of local practice (E10).

Access to PGx practice sites for laboratory or experiential learning poses an additional challenge, especially in countries where PGx services are limited or not yet implemented (E3, E8, E12, and E16). This restricts opportunities for hands-on learning and requires educators to develop alternative strategies, such as simulations, virtual cases, or structured application-based activities, to ensure that students still gain practical exposure to PGx concepts.

## 4 Discussion

Pharmacists are globally recognized as critical players in PGx implementation and precision medicine, given their expertise in pharmacotherapy and medication optimization (Haga, 2023; Muzoriana et al., 2017). Consistent with international evidence, experts in this study affirmed that pharmacists should integrate PGx into their broader scope of practice and that PGx competence is essential for all pharmacists, regardless of their practice setting. However, as highlighted in global studies, pharmacists and pharmacy students often report poor PGx knowledge despite having positive attitudes and perceptions towards PGx, underscoring a persistent gap between perceived value and preparedness (Alexander et al., 2014; Alhaddad et al., 2022; Balaban et al., 2026; Dias et al., 2014; Ghazal et al., 2023; Hundertmark et al., 2020; Ibrahim et al., 2025; Rahma et al., 2020). Insufficient capacity, context-specific evidence-based guidelines, limited research, infrastructure, and cost of PGx testing have been identified as barriers to PGx implementation and sustainable practices (Elewa and Awaisu, 2019; Balaban et al., 2026; Tata et al., 2020; Twesigomwe et al., 2025). Although healthcare professionals realize the value of PGx, they feel inadequately prepared to implement PGx (Wu R. R. et al., 2024). A lack of PGx education is a global barrier to PGx implementation (Ibrahim et al., 2025); therefore, Nagy et al. (2020) call for urgent education for pharmacists to fulfill their potential in precision medicine.

### 4.1 The need for structured, competency-based pharmacogenomics education

Insights from experts emphasized the pharmacist’s role in applying PGx within evidence-based medicine practice. Experts strongly supported the inclusion of PGx as a core competency in pharmacy education, echoing concerns in the literature about insufficient exposure to critical PGx principles, appropriate test utilization, and ELSI considerations (Haga and Moaddeb, 2019). Existing PGx competency frameworks provide a foundation for standardization (Callier et al., 2014; Gammal et al., 2022; Giri et al., 2018; O' Brien et al., 2009; Roederer et al., 2017; Weitzel et al., 2016); yet, experts emphasized that competencies must evolve alongside the rapidly changing PGx landscape. A tiered, competency-based educational approach for PGx education emerged from the data. The authors, therefore, recommend an integrated competency-based framework to guide higher education institutions in scaffolding PGx learning from basic to advanced levels aligned with calls for global standardization (Formea, 2024). Balaban et al. (2026) support competency-based PGx training to address the educational gaps in PGx.

### 4.2 Approach to graduate pharmacy education

Balaban et al. (2026) recommend integrating PGx education into pharmacy training, expanding access to evidence-based PGx resources and clinical decision-support tools, and strengthening multidisciplinary collaboration to better prepare pharmacists for practice.

#### 4.2.1 Longitudinal and integrated pharmacogenomics curriculum design

Experts advocated for a longitudinal, integrated PGx-learning experience, consistent with recommendations from the literature (O' Brien et al., 2009; Wu J. Q. et al., 2024). This approach creates an opportunity to build on PGx knowledge and skills after graduate education through postgraduate training, research, and continuing education. A framework for the proposed longitudinal curriculum, however, does not exist (Wu J. Q. et al., 2024).

Experts agreed that pharmacy graduates should possess foundational PGx competencies, including the ability to educate health professionals, students, and patients; interpret PGx reports; and provide basic PGx counseling. Although the American College of Clinical Pharmacy has developed didactic curriculum toolkits to guide PharmD programs in delivering clinical education (Kolanczyk et al., 2024), PGx content is notably absent. This omission highlights a critical gap in current curriculum guidance and reinforces the need for dedicated, standardized PGx competencies globally to ensure that all pharmacy graduates are adequately prepared for emerging roles in precision medicine.

While standalone PGx courses offer depth and flexibility (Weitzel et al., 2016), integration across pharmacology, pharmacokinetics, and therapeutics enables repeated exposure and contextual application. Pharmacogenomics education is mostly offered as didactic integrated courses throughout graduate education, with assessments focused on foundational knowledge (Ibrahim et al., 2025) in the Middle Eastern and North African region. The variability in current PGx teaching hours (2 h–30 h across institutions) reflects the lack of consensus on optimal curricular time and reinforces the need for structured guidance. Gurwitz et al. (2005) recommend a minimum of 8 h of teaching as part of the basic pharmacology curricula.

#### 4.2.2 Application-based and experiential learning

Experts emphasized that PGx education must extend beyond didactic teaching to include application-based learning, higher-order thinking, and real-world clinical scenarios. Application-based activities were viewed as essential for developing the confidence and competence of pharmacists required to influence PGx-related decision making in clinical settings. Further higher-order thinking is critical to prepare pharmacist to navigate the complexity and evolving nature of PGx evidence. This aligns with evidence supporting simulation laboratories, case-based learning, and OSCEs as effective strategies for developing PGx counseling and critical decision-making skills (Lee et al., 2015; Wu R. R. et al., 2024). Personal genotyping was viewed as a powerful experiential tool, though its feasibility in resource-limited settings remains constrained by cost (Burghardt et al., 2021; Frick et al., 2016; Grace et al., 2021). However, paper-based clinical scenarios could be used as an alternative to apply PGx knowledge.

Experiential learning through practical experience opportunities, e.g., Advance Pharmacy Practice Experience or internships, was identified as ideal for translating theory into practice. However, experts from resource-constrained countries noted that limited PGx implementation restricts access to practice sites (E3, E12, and E16). Partnerships with healthcare institutions may help overcome these barriers by providing shared learning environments and access to PGx expertise.

#### 4.2.3 Community engagement and ethical, legal, and social considerations

It is evident from the results that education for health professions and the community should occur in parallel to build public trust and support the responsible uptake of PGx services. Community engagement emerged as an important yet context-dependent component of PGx education. Educating patients, the public, and professional colleagues about the benefits and limitations of PGx and providing counseling that is culturally sensitive and ethically informed are recognized as essential clinical PGx competencies (Academy of Science of South Africa, 2018; Gammal et al., 2022). This approach was viewed as essential for building public trust and supporting the responsible uptake of PGx services.

Experts agree that patient and public education is essential, particularly in settings where PGx is novel. This aligns with literature emphasizing the importance of community engagement in building trust, improving understanding of PGx results, and supporting ethical implementation (Nabukenya et al., 2024; Tindana et al., 2015). Incorporating ELSI topics into PGx education is, therefore, critical, especially in culturally diverse contexts. Claw et al. (2024) highlight four areas of focus for effective community engagement: engaging the community, increasing PGx knowledge of variation in the community, clinical implementation of PGx, and inclusion of traditional knowledge and practices. Integrating traditional knowledge and local community perspectives is particularly relevant for South Africa’s diverse population.

Roosan et al. (2021) propose PGx cascade testing (PhaCT) as a strategy to expand PGx knowledge among patients and improve medication-related outcomes. PhaCT involves identifying individuals who may carry clinically relevant PGx variants based on family history and testing results from related family members. This approach highlights the value of thorough evaluation of history and positions pharmacists to identify patients who may benefit from PGx testing and personalized therapy.

### 4.3 Continuing professional development and postgraduate pathways

Given the evolving nature of PGx, experts highlighted the importance of CE for practicing pharmacists. Various international organizations offer certificate courses to fulfill the need to educate practicing pharmacists (American College of Clinical Pharmacy, 2025; University of Florida, 2026; American Society of Health-System Pharmacists, 2026). This highlights the role of professional societies in providing CE opportunities for a global audience. Preferred CE modalities from the literature include workshops, self-directed learning, ward rounds, internships, short courses, and conferences (Albitar and Alchamat, 2021; Alhaddad et al., 2022; Alsaloumi et al., 2019; Bagher et al., 2021; Bannur et al., 2014; Karuna et al., 2020; Pop et al., 2022). For pharmacists seeking to perform advanced PGx services, postgraduate training through residencies, certificates, or PhD programs was viewed as essential for leading PGx implementation and managing complex cases.

Experts highlighted that residency training provides pharmacists with the advanced, practice-based competencies required to lead PGx implementation. In line with this, the American Society of Health-System Pharmacists has begun formalizing PGx residency standards, offering institutions structured guidance for developing specialized training pathways that prepare pharmacists for leadership responsibilities in precision medicine (American Society of Health-System Pharmacists, 2018).

### 4.4 Implications for South Africa and other resource-limited settings

Expertise and published experience in PGx remain concentrated in high-income countries, meaning that the evidence-base largely reflects contexts with greater resources and established research capacity. This underscores the urgent need for increased PGx research, education, and implementation insights from South Africa and other resource-limited settings to strengthen the global evidence-base for PGx. South Africa’s diverse population stands to benefit significantly from personalized medicine; yet, effective implementation requires a context-specific, competency-based PGx curriculum for health professionals, particularly pharmacists, aligned with local healthcare structures, resource constraints, and system readiness. Building faculty expertise, fostering partnerships with healthcare institutions, and adopting flexible and scalable teaching approaches are essential for preparing pharmacists for precision medicine healthcare. A standardized PGx curriculum framework developed with input from South African academics, local PGx experts, regulatory authorities, and accreditation bodies could enhance consistency, quality, and implementation readiness across pharmacy programs and offer a model for other resource-limited settings.

## 5 Limitations

The authors acknowledge that most participants were pharmacists from the developed countries, with limited African representation, which may bias the findings. Although the study focused on PGx education for pharmacists, the authors recommend including insights from the inter-professional team, including non-pharmacists, in future research to strengthen role clarity and guide curriculum development.

International societies were approached to identify experts, but responses predominantly came from developed countries, which is an expected outcome given the more advanced state of PGx in these settings. To ensure context-specific and locally relevant recommendations, perspectives from developing countries should be more intentionally included.

It is essential to acknowledge that the study findings and recommendations have not been reviewed, validated, or endorsed by South African curriculum developers or accreditation bodies to regulatory stakeholders. Further research is needed to inform the development of a proposed pharmacy curriculum that includes PGx.

## 6 Conclusion

International PGx experts highlighted the need for pharmacists to have clearly defined core competencies in PGx to contribute effectively to clinical practice. Integrating PGx as a required component of pharmacy education would help ensure that graduates develop the foundational knowledge and practical skills necessary to support PGx-informed care. A longitudinal, scaffolded learning experience designed and delivered by academics and health professionals with PGx expertise can further enable students to apply PGx principles in real-world settings. However, achieving meaningful and sustainable PGx education will depend on strong collaboration between higher education institutions and healthcare organizations to align the curriculum design, clinical training opportunities, and workforce needs. The research findings offer expert-informed recommendations that can guide the future development of PGx frameworks within pharmacy education.

## Data Availability

The raw data supporting the conclusions of this article will be made available by the authors, without undue reservation.
