# Supplementary material for: International expert perspectives on pharmacogenomics in pharmacy education: a qualitative study with implications for South Africa
Source: Front Pharmacol. 2026 Aug 18;17:1888534. doi: 10.3389/fphar.2026.1888534 (PMC13529995; doi:10.3389/fphar.2026.1888534)
Supplement: Supplementary file 1 [file Supplementaryfile1.docx]

Supplementary Material

# 1 Semi-structured interview guide

**Interview Schedule for Pharmacogenomics Experts**

In this interview I would like to determine your perspectives on including PGx in pharmacy education globally so that this information can help to inform development of a curriculum framework for PGx education for pharmacists in South Africa.

This interview is divided into the following sections: demographics, PGx in practice, role of higher education institutions and professional societies in PGx education, recommendations for PGx education for 1) undergraduate pharmacy education, 2) graduate/postgraduate pharmacy education, and 3) practicing pharmacists.

**Demographics**

1. Can you briefly introduce yourself, including your area of expertise in education and research in the area of PGx?
2. Can you elaborate on your education and training in PGx?

Prompt: Completion of Short courses/continuing educational opportunities/residency programmes

3. Are you part of any professional PGx organisation(s)?

If yes, which organisation and what is your role in this/these organisations?

4. Do you think pharmacists can benefit by being involved in global or local PGx organisations?

**Pharmacogenomics in practice**

5. How would you describe the pharmacist’s roles and responsibilities in

PGx education

PGx research

PGx clinical implementation

6. Research suggests that all pharmacists require a basic understanding of PGx and that advanced PGx education is offered to pharmacists for further specialisation or to perform more advanced roles. How would you differentiate between basic and advance PGx roles for pharmacists?
7. In your opinion, what knowledge and skills are necessary for pharmacists to work in clinical PGx implementation?
8. What advice/recommendations do you have for low to middle income countries to ensure successful PGx education for pharmacists?

**Role of higher education institutions and professional societies**

9. What do you think are the roles and responsibilities of higher education institutions in educating pharmacy students and practicing pharmacists on PGx?
10. What do you think are the roles and responsibilities of professional societies in educating practicing pharmacists on PGx?

**In the next section I want to explore your recommendations for PGx for undergraduate pharmacy education**

11. Where applicable, what are your recommendations for incorporating PGx education in undergraduate pharmacy education programme with regards to:
    a. Pedagogies/Approaches/Strategies for learning and teaching
a. Should PGx be offered as integrated or standalone modules and why?
b. Year levels at which PGx education should be offered
c. Content in terms of lecture topics
d. Activities for learning
e. Assessment approaches
f. Number of hours/credits for PGx focused education
g. Which disciplines should be involved in PGx education and why?
h. Considering experiential teaching, which type of experiential learning opportunities do you recommend for PGx education?
i. Do you have success with experiential learning opportunities? If yes, can you elaborate?
j. Do you think undergraduate students could benefit from doing PGx research projects?
k. Do you think students can benefit by being involved in community engagement (patient/public interaction) in PGx? What are your recommendations to incorporate community engagement in PGx education?

**In the next section I want to explore your recommendations for pharmacy graduates/postgraduate programmes**

12. Where applicable, what would be your recommendations for incorporating PGx education in postgraduate pharmacy education programmes, specifically a structured master’s programme in terms of:
a. Pedagogies/Approaches/Strategies for learning and teaching
b. Should PGx education be offered as integrated or standalone modules?
c. Content/lecture topics
d. Activities for learning
e. Assessment approaches
l. Number of hours/credits for PGx focused education
f. Disciplines/stakeholders to be involved and why
g. Considering experiential learning education, what would be requirements for experiential learning in pharmacy education?
h. Do you think graduate students could benefit from doing PGx research projects?
i. Do you think students can benefit by being involved in community engagement (patient/public interaction) in PGx? What are your recommendations to incorporate community engagement in PGx education?

**In the next section I want to explore your recommendations for practicing pharmacists**

13. Where applicable, what would be your recommendations for PGx education for pharmacists in practice?
    a. Type of education e.g. CPD/Short courses/ short learning courses
a. Pedagogies/Approaches/Strategies for learning and teaching
b. Content in terms of lecture topics
c. Activities for learning
d. Stakeholders to be involved in executing this education
e. Based on your experience, what strategies have been most effective to ensure integration of pharmacogenomics into clinical practice?

Follow up questions

What type of quality improvement projects have been successful to support PGx? How can patients/the public be incorporated to support PGx implementation?
